# Supplementary material for: Identification of photoperiod-induced specific miRNAs in the adrenal glands of Sunite sheep (Ovis aries)
Source: Front Vet Sci. 2022 Jul 22;9:888207. doi: 10.3389/fvets.2022.888207 (PMC9354845; doi:10.3389/fvets.2022.888207)
Supplement: Supplementary file 1 [file Data_Sheet_1.ZIP › Supplementary file/miRDeep2 file.zip!/miRDeep2 file/Novel_Structure/SPLP42A3/Novel_564.pdf]

Provisional ID	:	NC_019466.2_18663
Score total	:	6.8
Score for star read(s)	:	3.9
Score for read counts	:	0.6
Score for mfe	:	0.7
Score for randfold	:	1.6
Score for cons. seed	:	
Total read count	:	13
Mature read count	:	12
Loop read count	:	0
Star read count	:	1

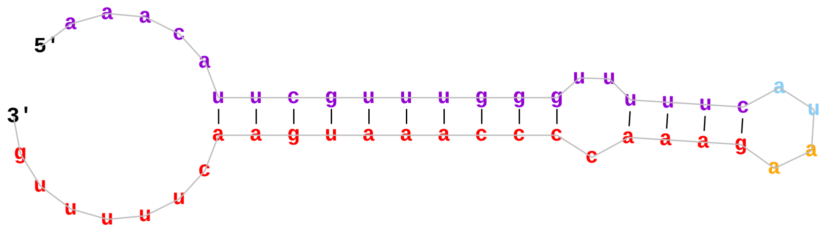

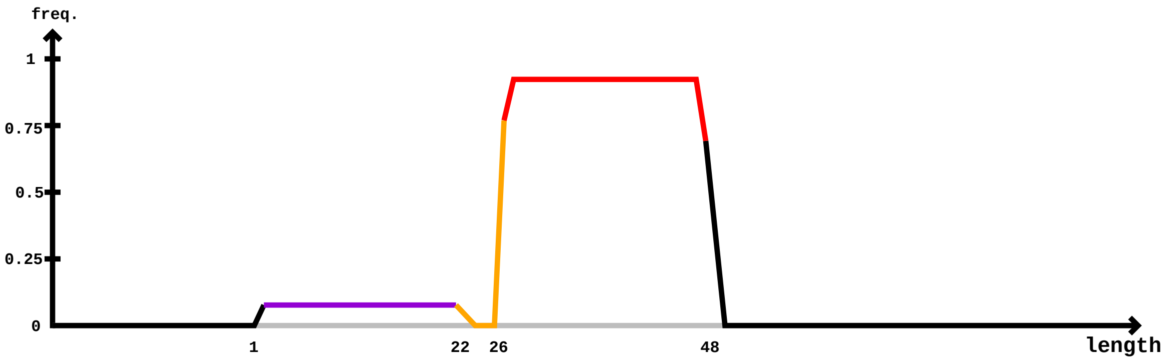


```
                         Star                     Mature
5'- aacaucuauugagcuggccaaaacauucguuuggguuuuucauaagaaaccccaaaugaacuuuuugucaaacccaauacaaucucaguggcuucucacauggcucagugg   -3'    obs
    aacaucuauugagcuggccaaaacauucguuuggguuuuucauaagaaaccccaaaugaacuuuuugucaaacccaauacaaucucaguggcuucucacauggcucagugg          exp
    .....((((((((((((.((((..((((((((((..((((....)))).))))))))))..))))))))..............(((((......))).))))))))))    reads     mm        sample
    ....................aaacauucguuuggguuuuuc......................................................................    1         0         seq
    .............................................gaaaAcccaaaugaacuuuuu.............................................    3         1         seq
    .............................................gaaaAcccaaaugaacuuuuug............................................    6         1         seq
    .............................................Aaaaccccaaaugaacuuuuug............................................    1         1         seq
    ..............................................aaaccUcaaaugaacuuuuug............................................    1         1         seq
    ..............................................aaaccccaaaugaacuuuuug............................................    1         0         seq
```
